# Non-Invasive Assessment of Metabolic Adaptation in Paediatric Patients Suffering from Type 1 Diabetes Mellitus

**DOI:** 10.3390/jcm8111797

**Published:** 2019-10-26

**Authors:** Phillip Trefz, Sibylle C. Schmidt, Pritam Sukul, Jochen K. Schubert, Wolfram Miekisch, Dagmar-Christiane Fischer

**Affiliations:** 1 Department of Anaesthesiology and Intensive Care Medicine, Rostock Medical Breath Research Analytics and Technologies (ROMBAT), Rostock University Medical Centre, 18057 Rostock, Germany; Pritam.Sukul@uni-rostock.de (P.S.); jochen.schubert@uni-rostock.de (J.K.S.); Wolfram.Miekisch@uni-rostock.de (W.M.); 2 Department of Paediatrics, Rostock University Medical Centre, 18057 Rostock, Germany; schmidt_sibylle@t-online.de (S.C.S.); dagmar-christiane.fischer@med.uni-rostock.de (D.-C.F.)

**Keywords:** type 1 diabetes mellitus, pediatrics, breath analysis, non-invasive, metabolic adaptation, PTR-ToF-MS, volatile biomarkers

## Abstract

An analysis of exhaled volatile organic compounds (VOC) may deliver systemic information quicker than available invasive techniques. Metabolic aberrations in pediatric type 1 diabetes (T1DM) are of high clinical importance and could be addressed via breathomics. Real-time breath analysis was combined with continuous glucose monitoring (CGM) and blood tests in children suffering from T1DM and age-matched healthy controls in a highly standardized setting. CGM and breath-resolved VOC analysis were performed every 5 minutes for 9 hours and blood was sampled at pre-defined time points. Per participant (*n* = 44) food intake and physical activity were identical and a total of 22 blood samples and 93 minutes of breath samples were investigated. The inter-individual variability of glucose, insulin, glucagon, leptin, and soluble leptin receptor relative to food intake differed distinctly between patients and controls. In T1DM patients, the exhaled amounts of acetone, 2-propanol, and pentanal correlated to glucose concentrations. Of note, the strength of these correlations strongly depended on the interval between food intake and breath sampling. Our data suggests that metabolic adaptation through postprandial hyperglycemia and related oxidative stress is immediately reflected in exhaled breath VOC concentrations. Clinical translations of our findings may enable point-of-care applicability of online breath analysis towards personalized medicine.

## 1. Introduction

Through recent advances in human medicine, the immediate assessment of disease-driven metabolic effects and variations has gained clinical significance, especially for metabolic disorders such as diabetes which are large contributors to global disease burdens. About 86,000 children per year are diagnosed with type 1 diabetes (T1DM) [1,2,3]. T1DM is caused by the irreversible and progressive destruction of pancreatic ß-cells, finally leading to a completely missing secretion of insulin [4]. As a consequence, a life-long administration of insulin is required to ensure near-normal metabolism of glucose, i.e., to prevent hyperglycemia. However, insulin monotherapy is hardly able to restore metabolic homeostasis and the interplay with leptin and glucagon as the glucoregulatory partner of insulin [5,6,7]. This might explain at least in part why T1DM patients are at high risk to develop serious comorbidities at a young age [8,9]. 

Despite all efforts to optimize insulin supply, most of the T1DM patients suffer from recurrent hyperglycemic episodes. Hyperglycemia has been related to (i) an increased polyol pathway flux, (ii) an increased formation of advanced glycation end products (AGE), (iii) an activation of protein kinase C isoforms, and (iv) an increased hexosamine pathway flux. Either of these mechanisms lead to the formation of superoxide by the mitochondrial electron transport chain. Hyperglycemia and glycemic variability are thus thought to induce oxidative stress and the latter is envisaged as the main culprit of macro- and microvascular disease and/or diabetic neuropathy [10,11,12,13,14,15,16,17,18]. Therefore, reliable long-term metabolic control might be the best measure to prevent such comorbidities. Apart from the determination of HbA_1C_ as an established marker of long-term glycemic control and the discontinuous determination of blood glucose for a targeted administration of insulin, continuous glucose monitoring (CGM) enables a detailed assessment of glycemic variability via the measurement of interstitial glucose concentration in time intervals of only a few minutes. However, CGM is not suited for monitoring the induction of oxidative stress. As repeated invasive analysis is not applicable to routine medical practice, especially in pediatric patients, non-invasive methods are necessary for the analysis of metabolites originating from the activation of alternative metabolic pathways. 

Real-time analysis of volatile organic compounds (VOCs) in exhaled breath is quick, point-of-care applicable, and most importantly, non-invasive [19]. In general, hundreds of VOCs are detectable in trace amounts (parts per trillion by volume to parts per billion by volume (pptV–ppbV)) in human breath under different physiological and pathophysiological conditions [20,21,22,23,24,25,26,27,28]. As VOCs are exhaled shortly after their production, they may deliver metabolic insight quicker than conventional invasive techniques. 

Breath analysis might enable the assessment of metabolic adaptation driven by glycemic variability as volatile biomarkers indicating oxidative and metabolic stress are detectable in exhaled breath even within minutes after their production [29,30,31]. Direct real-time mass spectrometric techniques such as proton-transfer-reaction-time-of-flight mass spectrometry (PTR-ToF-MS) or selected-ion-flow-tube mass spectrometry (SIFT-MS) enable the detection of fast changes without requiring additional sample preparation [32,33,34,35]. Breath analysis in combination with CGM thus may provide information beyond conventional analysis. 

## 2. Patients and Methods

### 2.1. Study Design and Participants

**Figure 1:**
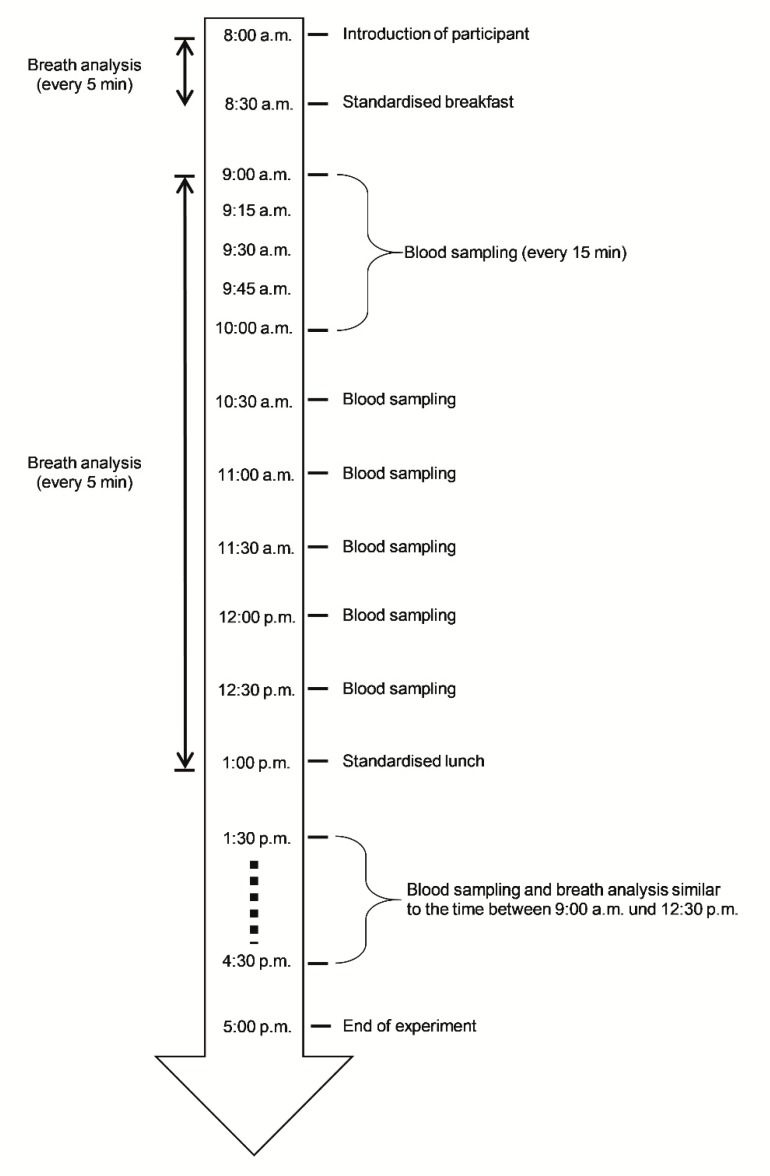
Representation of the study design. Insertion of continuous glucose monitoring (CGM) took place the evening before and placement of an indwelling venous canulae was done on the morning before breakfast. Total number of blood samples was 22 and at least 93 breath samples were measured per participant. At the times indicated, blood was drawn for preparation of plasma.

The study was approved by the institutional Ethics Committee (University Medical Centre Rostock, Rostock, Germany) in accordance with the Declaration of Helsinki (approval number: A 2012 0103). All subjects and their parents gave their written and informed consent prior to participation. Pediatric patients aged between 12 and 16 years, suffering from type 1 diabetes mellitus for at least 2 years and being treated at our institution were eligible for participation in this cross-sectional study. Additional inclusion criteria were stable therapeutic regimen with either multiple daily insulin injections (MDII) or continuous subcutaneous insulin infusions (CSII, pump therapy) and a concentration of C-peptide below 0.3 nmol/L. Both healthy controls and patients were excluded if they had any febrile illness within the preceding 3 months, chronic inflammatory-/rheumatic disease (e.g., Crohn’s disease, rheumatoid arthritis), hepatitis, HIV, glucocorticoid treatment, liver-, renal-, or cardiac failure, or hereditary dyslipidemia. Healthy controls matched for age and sex were recruited among relatives and friends of the patients and both were instrumented for blinded CGM (iPro2 Professional CGM, Medtronic GmbH, Meerbusch, Germany) the day before the start of the study examinations [36]. The time schedule of the study is presented in Figure 1 and aside from standardized food intake at breakfast and lunch, participants had unlimited access to water throughout the study. Real-time breath analysis by means of PTR-ToF-MS and masked CGM were synchronized throughout the experiment and data was recorded every 5 minutes over a period of 9 hours from 08:00 to 17:00. During the 30 minutes of breakfast and lunch, no breath analysis was performed. A minimum of 93 breath samples per volunteer was obtained in this way. Blood samples were taken before breakfast and then every 15 minutes during the first hour after breakfast, the first hour after lunch, and every 30 minutes during the remaining hours. This resulted in a total of 22 blood samples for each participant. 

### 2.2. Procedures 

Demographic and clinical data were gathered by interview and/or chart review. Individual age- and gender-related SD scores (SDS) for height, weight, and BMI were calculated as described by Kromeyer-Hauschild et al. [37]. Serum and EDTA-plasma were aliquoted and stored at −80 °C until further analysis, while blood glucose was determined immediately by established automated procedures at the Department of Clinical Chemistry and Laboratory Medicine, Rostock University Medical Center. The concentration of triglycerides and cholesterol were determined manually (DiaSys Diagnostic Systems GmbH, Holzheim, Deutschland). For quantitative assessment of insulin (Iso-Insulin ELISA, Mercodia AB, Uppsala, Schweden), glucagon (Glucagon Quantikine ELISA, R&D Systems, Inc., Minneapolis, MN, USA), leptin (Human Leptin ELISA, TECOmedical AG, Sissach, Schweiz), and the soluble leptin receptor (sLepR, Human Leptin Receptor ELISA, BioVendor LM a. s., Brno, Czech Republic) enzyme immunoassays were used essentially as described by the manufactures and all samples were measured in duplicate. Results were normalized to the value obtained before breakfast and/or lunch as indicated. 

### 2.3. Breath Analysis

PTR-ToF-MS sampling, measurement, and data analysis have been described before [33]. Briefly, breath was sampled continuously in side-stream mode by means of a heated 6 m long silcosteel transfer line while the participant was breathing evenly through a sterile mouthpiece. The mouthpiece did not introduce any breathing resistance and sampling was done in a resting seated position [35]. One minute of breath was analyzed like this in 5-minute intervals. A PTR-ToF-MS 8000 (Ionicon Analytik GmbH, Innsbruck, Austria) was used in the study. 

The PTR was set to a time resolution of 200 ms and a sampling flow of 20 mL/min. The drift voltage was 610 V, the drift temperature was 75 °C, and the drift tube pressure was 2.3 mbar, resulting in an E/N ratio of 138 Td. Mass scale was recalibrated after every run of 60 s. Masses used for that purpose were 21.023 (H_3_O^+^-Isotope), 29.998 (NO^+^), and 59.049 (protonated acetone). Expiratory and inspiratory phases were recognized by means of the “breath tracker” algorithm [33] and acetone was used as the tracker substance. 

Within this study we focused on VOCs with a known or postulated relation to T1DM or metabolic processes that may be relevant for T1DM. Only VOCs with a signal-to-noise ratio of at least 3 (noise was determined via blank measurements) and a higher abundance in expiration compared to inspiration and differences between groups or time points were considered as potential marker substances. Expiratory abundance had to be above inspiratory abundance plus the standard deviation of inspiratory abundance. For quantification of VOCs, calibrations with pure reference substances and adapted sample humidity using a liquid calibration unit (LCU, Ionicon Analytik GmbH, Innsbruck, Austria) were performed essentially as described before [38]. 

### 2.4. Statistical Analysis

Sigma Plot 14 (Systat Software GmbH, Erkrath, Germany) and SPSS 17 (IBM Software, Armonk, USA) were used for graphic presentation and statistical analysis. Repeated-measurement ANOVA on ranks (Friedman repeated measures analysis of variance on ranks, Shapiro–Wilk test for normal distribution, and Dunn’s post hoc method for pairwise multiple comparisons between all groups; *p*-value ≤0.05 was considered significant) was performed via Sigma Plot 14. For the Pearson product moment correlation analysis, SPSS 17 was used.

For an assessment of longitudinal changes of analytes determined in plasma and breath, concentrations measured in samples obtained throughout the morning (after intake of breakfast until 5 min before lunch) and during the afternoon (after intake of lunch until the end of the examination period) were normalized to the concentrations determined right before intake of the meal. Dunn’s method was used for multiple comparisons versus control. A *p*-value of 0.05 or lower was considered statistically significant.

Concentration differences between healthy controls and T1DM patients were evaluated for each time point via Mann–Whitney rank sum tests. A *p*-value of 0.05 or lower was considered statistically significant.

## 3. Results

A total of 44 participants (22 T1DM patients) were enrolled and underwent CGM, direct real-time PTR-ToF-MS for breath analysis, and blood sampling in a highly standardized setting and during a 9-hour examination period. The time schedule is outlined in Figure 1 and an overview on the clinical and anthropometric data is given in Table 1. A total of 12 and 10 patients used multiple daily injections and continuous subcutaneous insulin infusion, respectively. Per participant, a total of 22 blood samples was analyzed and more than 90 breath samples were taken. 

### 3.1. Continuous Glucose Monitoring

**Figure 2:**
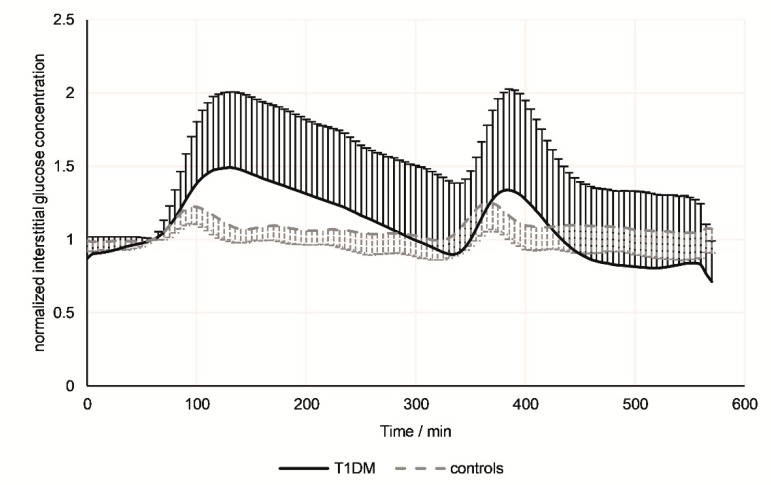
Time resolved glucose monitoring. Continuous glucose monitoring in healthy controls (empty squares; *n* = 22) and pediatric T1DM patients (full dots; *n* = 21) over the course of nine hours. Red lines indicate the intake of standardized meals (breakfast and lunch). Data was normalized to minute 60 (baseline before breakfast) to emphasize relative changes and minimize inter-individual variation.

**Figure 3:**
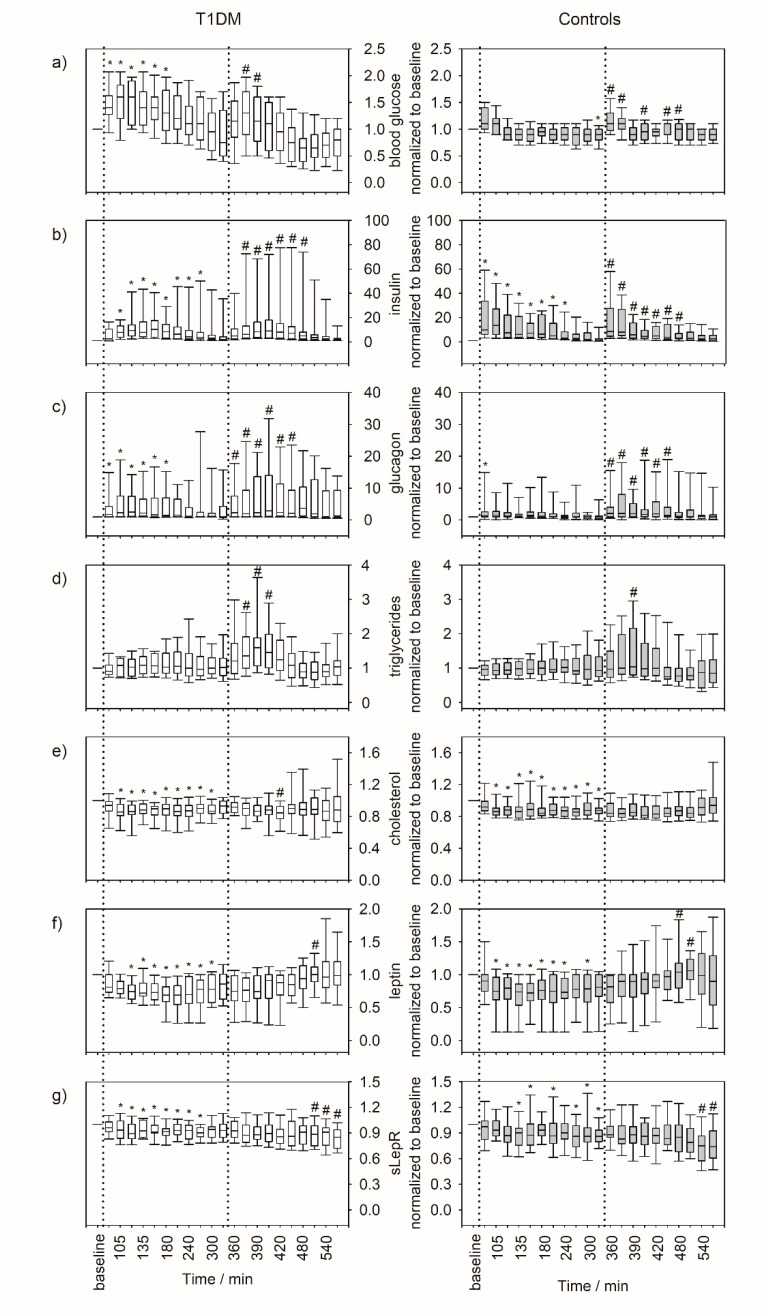
Box plots of serum parameters over the course of nine hours. (**a**) Blood glucose, (**b**) insulin, (**c**) glucagon, (**d**) triglycerides, (**e**) cholesterol, (**f**) leptin, and (**g**) sLepR. The graphs on the left show the course over of the nine hours of the experiment for T1DM patients (*n* = 22), the graphs on the right show the corresponding data from healthy controls (*n* = 22). Data were normalized to the baseline value (before breakfast) to emphasize relative changes and minimize inter-individual variation. Lunch was taken after the measurement at the 330th minute. Dotted lines indicate the intake of standardized meals. The asterisks (*) indicate statistically significant changes versus baseline (before breakfast) and hashes (#) indicate statistically significant changes versus the 330th minute (last measurement before lunch).

The results from continuous glucose monitoring throughout the 9-hour examination period are presented in Figure 2. Albeit the interstitial glucose concentrations increased after both meals, this was more pronounced and the inter-individual variation was higher in T1DM patients compared to controls. While in healthy controls, interstitial glucose concentrations returned to the baseline within 40 minutes after each meal, in T1DM patients a prolongated and delayed decrease was noted, especially after breakfast. The same changes over time and relative to the intake of meals was seen for blood glucose (Figure 3a). In general, blood glucose concentrations were significantly higher in the T1DM group compared to the control group (up to three-fold). Differences between the groups were most pronounced in postprandial phases and became smaller towards the end of the experiment (Table 2).

### 3.2. Serum Parameters

Box plots reflecting the normalized concentration of blood glucose, insulin, glucagon, triglycerides, cholesterol, leptin, and soluble leptin receptor (sLepR) throughout the examination period together with the results of the longitudinal statistical analysis are presented in Figure 3 and Table 2, respectively. Results from cross-sectional statistical comparisons are shown in Table S1.

Although insulin concentrations significantly increased in both groups after each meal (Figure 3b), the trend over time differed slightly. In controls, insulin concentrations peaked immediately after each meal and steadily declined afterwards. By contrast, and most probably due to the administration of exogeneous insulin, the increase and decrease was slower in T1DM patients. Furthermore, insulin concentration ranges were between 10.2–148.58 mU/L and 6.15–68.9 mU/L in T1DM patients and controls, respectively (Table 2).

In T1DM patients, glucagon concentrations peaked significantly after each meal and, similar to their blood glucose, were partially elevated in comparison to healthy controls. These differences were most pronounced following meals (and especially after lunch), when food intake induced a glucagon peak in T1DM patients but not in controls (Figure 3c). 

Although the diurnal profiles of triglycerides and cholesterol showed subtle differences between patients and controls with slightly elevated concentrations in the T1DM group, the overall concentration ranges were rather comparable in both groups (Figure 3d,e, Table 2). 

In patients and controls, leptin concentrations significantly decreased after breakfast and returned to initial concentrations around lunch time (Figure 3f). Leptin concentration measured during the morning tended to be higher in T1DM patients compared to controls but were in the same range in both groups after lunch (Table 2). Although the concentration of the sLepR was about two-fold higher in T1DM patients compared to controls (each p < 0.05), the diurnal profiles were fairly comparable in both groups (Table 2, Figure 3g).

### 3.3. Breath VOCs 

**Figure 4:**
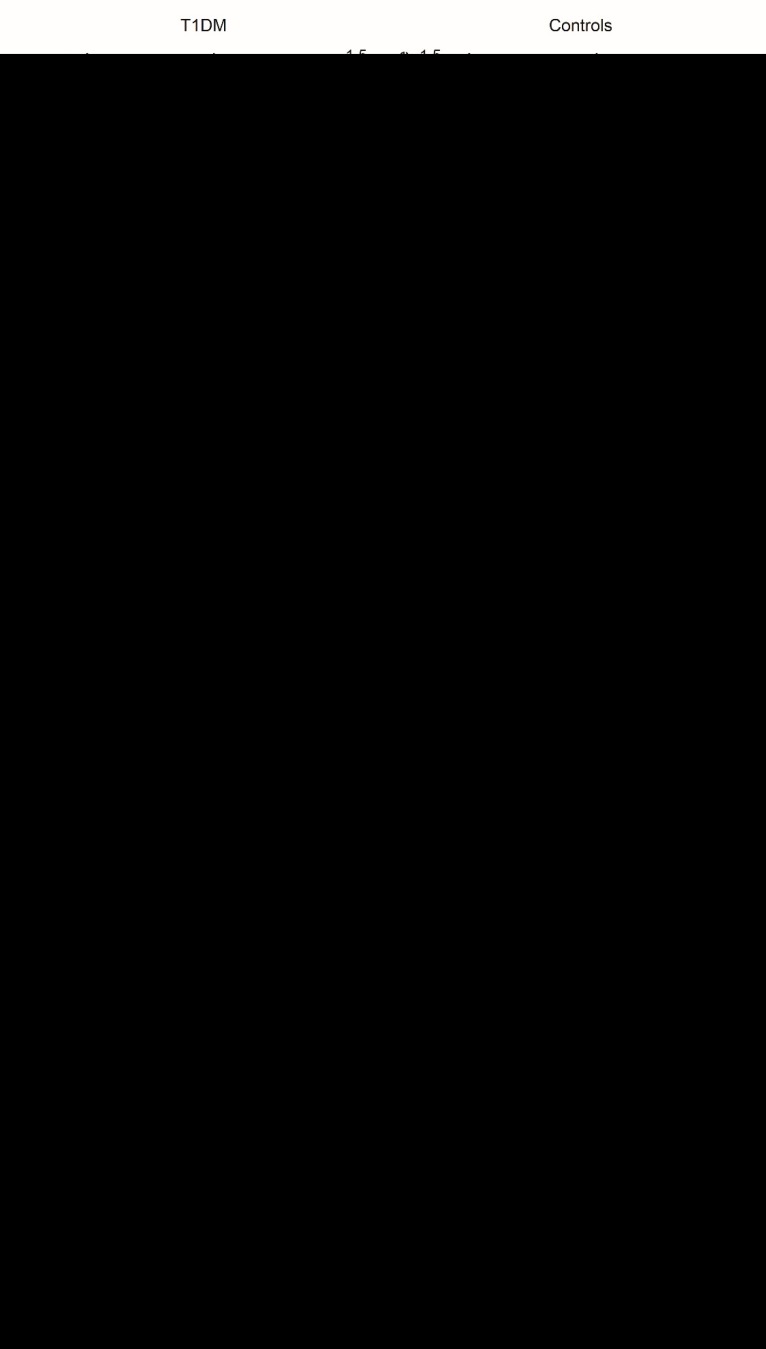
Box plots of breath VOCs over a course of nine hours. (**a**) Acetone, (**b**) 2-propanol, (**c**) pentanal, (**d**) ethanol, (**e**) dimethyl sulfide, (**f**) isoprene, and (**g**) limonene. The graphs on the left show the course over of the nine hours of the experiment for T1DM patients (*n* = 22), the graphs on the right show the corresponding data from healthy controls (*n* = 22). Data were normalized to the baseline value (before breakfast) to emphasize relative changes and minimize inter-individual variation. Lunch was taken after the measurement at the 330th minute. Dotted lines indicate intake of standardized meals. Asterisks (*) indicate statistically significant changes versus baseline (before breakfast) and hashes (#) indicate statistically significant changes versus the 330th minute (last measurement before lunch).

We focused the analysis of VOC data on acetone, 2-propanol, pentanal, ethanol, dimethyl sulfide, isoprene, and limonene as these compounds have been linked to glucose metabolism and/or to T1DM-related comorbidities including oxidative stress. Box plots of breath VOCs at the time points of blood sampling are presented in Figure 4 while the results of the longitudinal investigation of the data together with the concentration ranges are given in Table 3. Results from cross-sectional statistical comparisons are shown in Table S2. 

Acetone (Figure 4a) concentrations increased after breakfast and tended to decrease throughout the duration of the experiment. However, the initial increase was not statistically significant, while the decreasing trend reached statistical significance within one to three hours after each meal in both groups. This trend was more pronounced in T1DM patients and, similar to blood glucose levels, acetone concentrations were significantly (almost up to three-fold) higher compared to controls (Table 3). Differences between the groups were most pronounced following meals and became smaller towards the end of the experiment.

*2-propanol* (isopropanol, Figure 4b) concentrations tended to increase after breakfast and then slowly decreased over time (Figure 4 b). In either group, only the last measurements before lunch as well as those before the end of the examination period differed significantly from the pre-meal control values. 2-propanol concentrations were up to two times higher in T1DM patients compared to controls (*p* < 0.05, Table 3). Similar to acetone concentrations, differences between the groups became smaller towards the end of the experiment. 

In T1DM patients, the trend of pentanal (Figure 4c) was fairly comparable to that of acetone. In healthy controls, pentanal decreased after breakfast, increased slightly after lunch, and dropped again to pre-lunch levels. Apart from this and similar to acetone and 2-propanol, pentanal concentrations were significantly higher in the T1DM group and differences between the groups declined towards the end of the examination period (Table 3). 

In both groups, ethanol (Figure 4d) showed a significant increase 45 minutes after breakfast followed by a decrease back to initial levels and a more pronounced and immediate significant increase after lunch. This was followed by a decrease back to baseline after approximately one hour. Concentration ranges were comparable in the first half of the experiment, while at the end of the experiment ethanol concentrations were higher in T1DM patients (Table 3). 

Dimethyl sulfide (Figure 4e and Table 3) concentrations were comparable in patients and controls. In either group, a minor decrease after breakfast was followed by a significant increase towards the end of the examination period. However, the initial decrease was only statistically significant in the control group. 

Isoprene (Figure 4f) concentrations slightly decreased and then increased again after each meal. Concentration ranges were fairly comparable in both groups and concentrations were generally higher during the afternoon compared to the morning (Table 3). 

Limonene (Figure 4g) concentrations showed a major significant increase directly after breakfast in both groups followed by a decrease back to baseline levels within the next 45 minutes. Concentrations were in the same range in both groups (Table 3). 

### 3.4. Correlations Between Breath VOCs and Either Serum Parameters or CGM Data

Pearson product moment correlation analysis was performed to test for linear correlations between breath VOCs and either blood parameters or CGM data. Correlations were calculated for all data (T1DM patients and controls combined) as well as for either group separately. An excerpt of the results is given in Table 4 and a full overview can be found in Tables S3 and S4. 

The combined results from all participants revealed significant correlations between sLepR and either acetone, 2-propanol, and pentanal. However, this turned out to be driven mainly by data from controls. By contrast, the correlation between either one of the aforementioned VOCs and glucose was more pronounced in T1DM patients and almost identical for blood glucose and CGM data.

**Figure 5:**
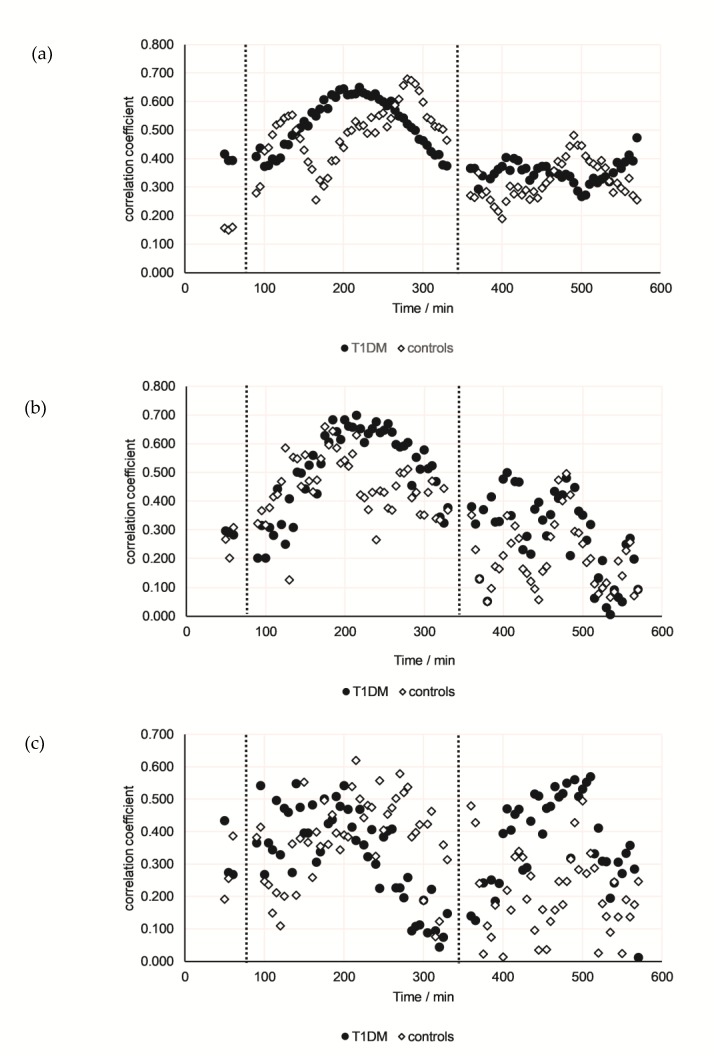
Time resolved correlations between acetone and interstitial glucose concentration (correlation coefficient versus time, 95 correlations in total). Dotted lines indicate intake of standardized meals. Full dots: T1DM patients (*n* = 21), empty squares: Healthy controls (*n* = 22); (**a**) acetone; (**b**) 2-propanol; and (**c**) pentanal.

Finally, for either group the correlation between glucose and either acetone, 2-propanol, or pentanal was calculated for every time point and the correlation coefficients were plotted as a function of time (*n* = 22 for control group and *n* = 21 for the T1DM group) (Figure 5). This approach indicated that the time of examination relative to the intake of meals and/or the time of the day had considerable impact. This effect is rather impressive for acetone and for this compound clear differences between patients and controls are visible. The correlation coefficients for 2-propanol and glucose showed a trend comparable to that of acetone, although differences between T1DM patients and controls were less pronounced. By contrast, in T1DM patients the correlation coefficients between pentanal and glucose increased after both meals, while no clear tendency was found for the control group. 

## 4. Discussion

Within this study, we used real-time CGM (rtCGM) in combination with a serial analysis of blood and exhaled breath in a highly standardized setting in pediatric T1DM patients and healthy controls over a time course of nine hours. We accumulated more than 4000 measurements of breath VOCs and interstitial glucose and almost 1000 blood samples from 44 participants. Standardization with respect to physical activity, the interval between meals, the type and amount of nutrients, and the restriction to alveolar breath sampling allows the generation of highly comparable diurnal profiles of breath and blood born parameters relative to the metabolic state. For real-time breath analysis, a PTR-ToF-MS was used and we focused on the exhalation of ethanol, acetone, isopropanol, dimethyl sulfide, isoprene, pentanal, and limonene. These compounds have been linked to glucose metabolism and/or to T1DM-related comorbidities including oxidative stress. Thus, we investigated the fade of classical markers via repetitive blood sampling and looked in parallel for the VOCs mentioned above. 

In the case of T1DM, acetone is certainly the most prominent VOC in breath and has previously been investigated under various conditions [39,40,41,42,43,44]. Acetone originates from enzymatic or non-enzymatic decarboxylation of acetoacetate [45] via Acetyl-CoA which is derived from pyruvate. Since the latter can originate from both glycolysis and lipolysis, it is impossible to differentiate the amount from either pathway. Regardless of this, we and others noted the significantly elevated exhalation of acetone in T1DM patients compared to controls [39,41,43,46,47], as well as the elevated blood levels in T1DM patients [48]. However, although the concentrations of glucose and acetone correlated in T1DM patients, the time and/or the interval between food intake, and breath sampling appears to be an important confounder. In the morning, when ketogenesis is high due to overnight fasting, correlations were low, most probably due to an additive effect of lipolysis and glycolysis on the formation of acetone. Correlation improved markedly after a moderate intake of carbohydrates and insulin-related inhibition of lipolysis. In the afternoon, correlation between acetone and blood glucose remained low. This was most probably due to postprandial hyperglycemia. Thus, a correlation of acetone with blood glucose is difficult even under healthy conditions and the time-resolved calculation of the correlation coefficients in our study strengthen this observation. Furthermore, the impact of hepatic alcohol dehydrogenase, i.e., the reduction of acetone to isopropanol, has to be considered [49]. In fact, T1DM patients exhaled significantly more isopropanol than their healthy peers and both, the diurnal profile as well as the time resolved correlation coefficients, closely resembled that of acetone. Thus, activation of this pathway in T1DM patients may attribute to acetone elimination and indicate metabolic adaptation. 

The shape of the normalized diurnal profiles of glucose, insulin, glucagon, lipids, leptin, and slepR are fairly comparable in T1DM patients and healthy controls, but differ with respect to slope and inter-individual variability, i.e., changes over time are faster in controls while inter-individual variability is much greater in T1DM patients. This might, at least in part, be due to the limited abilities of pharmaceutical insulin preparations to mimic the physiological interplay between insulin-induced cellular uptake of glucose and glucagon secretion. Under physiological conditions, insulin is secreted in response to increasing blood glucose levels and by inducing cellular glucose uptake and inhibition of pancreatic glucagon secretion at the same time it contributes to maintenance of glucose homeostasis [50]. Thus, in healthy individuals, glucagon concentration in blood remains constant as long as the blood glucose concentration stays within the normal range and insulin concentrations peak after meals. In contrast, in T1DM patients glucagon concentrations remain partially elevated even after meals and most likely aggravate postprandial hyperglycemia [50,51,52]. The latter, especially in concert with dyslipidemia is considered as a relevant contributor to the overall increased risk for the development of cardiovascular disease. 

On the one hand, poor glycemic control is likely to result in elevated cholesterol and triglyceride levels [53,54,55], on the other hand, peripheral hyperinsulinemia secondary to subcutaneous insulin administration contribute to qualitative abnormalities of lipid metabolism [54] as well and this has recently been shown in young T1DM patients [56]. Albeit no clear correlation between breath ethanol and serum triglycerides exists, the concentration profiles were quite similar, especially in the second half of the examination period, i.e., after challenge with carbohydrates (pasta) at lunch. Endogenous ethanol is supposed to originate from intestinal bacterial activity via fermentation of carbohydrates [57], i.e., breath ethanol increase is due to the intake with subsequent digestion of carbohydrates and the intestinal microbiota. While the carbohydrate intake at lunch was quite similar in both groups, postprandial hyperglycemia was not and even subtle differences with respect to the intestinal microbiota between T1DM patients and controls have to be considered. Altogether, this may have caused a slightly more pronounced increase of postprandial ethanol with higher peak ethanol concentrations in T1DM patients [28]. Elevated endogenous ethanol was also already previously detected in diabetic patients [27,58]. 

By contrast the exhalation of dimethyl sulfide, i.e., a breakdown product of methionine and generated mainly via bacterial activity in the gut, is rather identical in T1DM patients and controls [59,60,61]. Similarly, the diurnal profiles of the mono-terpenes isoprene and limonene were almost identical in T1DM patients and controls. Isoprene, a by-product of the cholesterol biosynthesis, is preferentially stored in fatty tissue and exhalation is largely dependent on physiological parameters [62,63]. In fact, isoprene exhalation can immediately change with changing ventilatory or hemodynamic parameters [64,65,66] and pronounced short term changes can serve as an indicator of physiological activity. Since the isoprene concentrations were fairly constant throughout the study, we can be certain that our results are not biased by physiological variation. Limonene is typically used as a fragrance in cleaning agents or food and thus almost always of exogenous origin if detected in exhaled breath. Nevertheless, Fernández del Rio and O’Hara et al. suggested limonene as a molecular probe for the non-invasive assessment of liver function [67,68]. Although liver function can be impaired in T1DM patients, the simultaneous and rapid increase of exhaled limonene immediately after breakfast is certainly due to the ingestion of orange juice [69]. 

On the long run, hyperglycemia is considered as the driving force of diabetic complications via stimulating the flux of the polyol and hexosamine pathway, the formation of advanced glycation end-product (AGE), and activation of protein kinase C (PKC) isoforms. While the increased polyol pathway flux might contribute to the formation of acetone and isopropanol, the overproduction of superoxide is viewed as the main culprit of the hyperglycemia related pathogenic mechanisms [70]. Superoxide is not only a cause of cell membrane damage via the peroxidation of lipids, but might translate into the formation of aldehydes, which in turn are eliminated via alveolar breath. In favor of this hypothesis, T1DM patients exhaled higher amounts of pentanal than healthy controls. Furthermore, the time resolved correlation between pentanal and blood glucose is quite similar to those observed for acetone or isopropanol and for either one of these VOCs the correlation with glucose is more pronounced in T1DM patients compared to controls. The tendency towards an increasing correlation coefficient in T1DM patients might indicate the induction of oxidative stress via the meal-related induction of hyperglycemia. 

The adipocyte hormone leptin and its plasma receptors (LepRs) play a crucial role in the regulation of appetite and energy expenditure through control of the insulin-glucose axis [71]. In healthy individuals, leptin leads to a decrease in insulin secretion [72], while insulin stimulates leptin secretion from adipose tissue. This hormonal regulatory feedback loop is probably disturbed in T1DM patients, i.e., the release of insulin is a matter of the pharmaceutical properties rather than under the control of leptin. However, the leptin concentrations were fairly similar in both groups and showed the expected postprandial decrease after breakfast, but not after lunch, and increased again towards the end of the study [73].

In T1DM patients, the concentration of the sLepR significantly decreased after lunch, while in healthy controls this occurred only in the last hour of the study. Leptin receptor concentrations were also significantly higher in T1DM patients, which is in agreement with the findings of Kratzsch et al. [73]. 

In summary, all of these parameters are in line with hyperglycemia as the main driving force behind metabolic adaptation and point to the induction of oxidative stress via several pathways and this is immediately reflected in exhaled breath VOC concentrations. 

## 5. Conclusions

Within this study we found distinct concentration changes of serum parameters and breath VOCs that could be linked to metabolic processes. Oxygenated compounds in exhaled breath such as acetone, 2-propanol, and pentanal showed significant correlations to blood glucose and interstitial glucose concentrations in the T1DM group, but much less pronounced correlations in the healthy control group. Of note, the strength of the correlation depends on the time of sampling, i.e., the interval between food intake and sampling of breath which explains the inconsistency within the literature on this issue. Our data strongly suggests that metabolic adaptation through postprandial hyperglycemia and related oxidative stress is immediately reflected in exhaled breath VOC concentrations. While an analysis of breath VOCs does not provide a robust assessment of blood glucose levels or an alternative to CGM, continuous monitoring of exhaled VOCs may offer non-invasive insights into T1DM-related metabolic adaptation even early in the progression of the disease. Once non-invasive markers are established, technological developments that replace complex analytical methods with simpler and cost-effect devices, such as sensor systems, are mandatory to enable a broad clinical application. 

If these limitations are considered, breath VOC analysis represents a unique monitoring tool in translational research due to the unlimited availability, dynamic occurrence, immediate reaction to metabolic adaptation, and its non-invasive nature. Once translated into a clinical perspective, this knowledge can not only apprehend the underlying pathophysiology of diabetes and similar metabolic disorders but may pave the path for breath analysis towards personalized medicine.

## Figures and Tables

**Table 1:** Study population: Dietary, therapeutic, and demographic data (median and range).

	Controls (13m/9f)	T1DM (Type 1 Diabetes) Patients (11m/11f)
Age [years]	14.2 (11.9–16.6)	14.2 (11.9–17.1)
Anthropometric data		
**Height**		
absolute [cm]	168.0 (143.4–187.6)	163.7 (146.8–184.3)
normalized [Z-Score}	0.27 (−1.43–2.30)	0.17 (–1.40–1.82)
**Weight**		
absolute [kg]	58.6 (32.8–58.6)	55.6 (36.7 - 96.2)
normalized [Z-Score]	0.15 (–1.86–1.68)	0.13 (–1.52–1.72)
BMI		
absolute [kg/m^2^]	20.3 (16–33.7)	21.7 (16.4–28.9)
normalized [Z-Score]	0.00 (−1.26–2.16)	0.6 (–1.30–1.63)
Carbohydrate intake with standardized meals [g/kg]	3.86 (2.43–5.87)	4.37 (2.42–6.98)
HbA_1c_		
at time of examination [%]	5.3 (4.8–5.9)	9.5 (6.3–11.5)
mean over the past year [%]		9.1 (6.4–11.6)
Duration of disease [years]	-	6.2 (2.3–11.4)
Duration of insulin therapy [years]	-	5.1 (1.9–10.8)
Daily dose of basal insulin [IU/kg]	-	0.37 (0.20–0.57)

**Table 2:** Time resolved analysis of the changes in serum concentrations. Per group and parameter, data for median is given, and significant changes (repeated measurement ANOVA on ranks in combination with Dunn’s method for pair-wise multiple comparisons versus control), are marked in bold and with an asterisk or hash, respectively. Significant changes in the first half of the experiment versus baseline (min 60, immediately before breakfast) is marked with an asterisk (*) and significant changes in the second half of the experiment versus minute 330 (immediately before lunch) are marked with a hash (#). *P*-values of 0.05 or lower were considered as significant.

			**T1DM Patients**				
**Time/min**	**Glucose /mmol/L**	**Insulin /mU/L**	**Glucagon /pg/mL**	**Triglycerides /mmol/L**	**Cholesterol /mmol/L**	**Leptin /ng/mL**	**sLepR /ng/mL**
**baseline**	9.85	10.16	19.15	0.50	3.10	7.43	30.20
90	14.85 *	30.14	99.82 *	0.46	2.91	5.95	31.39
105	16.00 *	70.41 *	96.39 *	0.51	2.73 *	6.08	30.51 *
120	16.10 *	90.24 *	81.36 *	0.52	2.61 *	5.15 *	30.02 *
135	14.25 *	105.26 *	81.76 *	0.55	2.73 *	5.38 *	27.91 *
150	14.35 *	110.32 *	78.21 *	0.60	2.67 *	4.91 *	31.23 *
180	14.00 *	85.20 *	79.43 *	0.62	2.73 *	4.49 *	28.28 *
210	12.40	80.49 *	76.89	0.63	2.73 *	4.20 *	28.87 *
240	12.30	57.29 *	74.97	0.52	2.69 *	4.40 *	28.90 *
270	12.15	56.42 *	75.63	0.52	2.83 *	4.67 *	28.42 *
300	10.40	45.35	68.94	0.51	2.80 *	5.38 *	29.37
**330**	8.95	34.75	66.21	0.55	2.87	5.33	30.06
360	11.90	48.43	**97.71 #**	0.63	2.97	5.00	32.19
375	**12.70 #**	**86.49 #**	**102.42 #**	**0.77 #**	2.82	5.46	32.04
390	**11.80 #**	**103.97 #**	**104.22 #**	**0.88 #**	2.71	5.01	30.23
405	10.25	**100.61 #**	**101.40 #**	**0.75 #**	2.68	4.81	28.62
420	10.05	**100.18 #**	**98.22 #**	0.64	**2.56 #**	5.62	28.13
450	7.90	**77.76 #**	**92.53 #**	0.51	2.89	5.07	28.82
480	6.85	**67.05 #**	82.33	0.43	2.79	5.81	28.94
510	6.90	46.59	80.75	0.43	2.84	**6.41** #	**29.71 #**
540	6.85	38.43	70.20	0.46	2.64	7.29	**27.94 #**
570	7.55	24.92	76.52	0.51	2.79	6.54	**26.48 #**
			**Healthy Controls**				
**Time/min**	**Glucose /mmol/L**	**Insulin /mU/L**	**glucagon /pg/mL**	**Triglycerides /mmol/L**	**Cholesterol /mmol/L**	**Leptin /ng/mL**	**sLepR /ng/mL**
**baseline**	5.00	6.16	37.75	0.42	2.47	4.27	17.53
90	5.90	45.49 *	73.03 *	0.38	2.29	3.68	16.67
105	5.65	52.51 *	62.63	0.40	2.32 *	2.93 *	15.26
120	4.75	38.64 *	66.61	0.38	2.29 *	2.80 *	15.43
135	4.65	32.23 *	59.03	0.35	2.21 *	2.84 *	14.16 *
150	4.55	30.43 *	62.74	0.36	2.27 *	2.98 *	15.94 *
180	4.75	28.10 *	62.79	0.37	2.12 *	3.04 *	16.76
210	4.70	25.41 *	59.37	0.36	2.32 *	2.32 *	14.45 *
240	4.40	20.63 *	41.26	0.37	2.08 *	2.94 *	14.76
270	4.70	18.36	58.83	0.33	2.08 *	2.96	12.95 *
300	4.65	13.94	6.37	0.39	2.22 *	2.81 *	13.82 *
**330**	4.35 *	9.21	20.82	0.42	2.13 *	3.00	15.21 *
360	**5.75 #**	**39.93 #**	**72.82 #**	0.40	2.09	3.05	15.11
375	**5.6 #**	**41.48 #**	**71.90 #**	0.45	2.07	3.37	14.98
390	4.65	**24.61 #**	**65.12 #**	**0.49 #**	2.26	3.65	14.78
405	**4.80 #**	**22.38 #**	**78.32 #**	0.46	2.08	3.32	14.81
420	4.80	**18.70 #**	**68.65 #**	0.45	2.12	3.40	14.18
450	**4.90 #**	**17.72 #**	**67.62 #**	0.35	2.21	3.78	14.35
480	**4.80 #**	**17.55 #**	66.12	0.34	2.34	**4.56 #**	13.96
510	4.60	14.37	52.96	0.30	2.28	**4.13 #**	14.04
540	4.60	14.03	53.27	0.32	2.42	3.58	**12.52 #**
570	4.40	11.43	35.45	0.36	2.49	2.62	**11.75 #**

**Table 3:** Time resolved analysis of volatile organic compounds (VOC) concentrations. Per group and parameter, data for median is given and significant changes (repeated measurement ANOVA on ranks in combination with Dunn’s method for pair-wise multiple comparisons versus control), are marked in bold and with an asterisk or hash, respectively. Significant changes in the first half of the experiment versus baseline (min 60, immediately before breakfast) are marked with an asterisk (*) and significant changes in the second half of the experiment versus minute 330 (immediately before lunch) are marked with a hash (#). P-values of 0.05 or lower were considered as significant.

			**T1DM Patients**				
**Time/min**	**Acetone /ppbV**	**2-Propanol /ppbV**	**Pentanal /ppbV**	**Ethanol /ppbV**	**Dimethyl Sulfide / ppbV**	**Isoprene /** **ppbV**	**limonene /** **ppbV**
**baseline**	824	1428	8.74	44.2	12.5	100.6	25.8
90	923	1275	7.05	72.2	11.4	107.5	48.0 *
105	891	1597	9.83	55.1	11.7	105.8	38.1 *
120	879	1533	7.81	64.2	11.4	88.5	36.5 *
135	846	1360	7.20	62.6 *	10.5	98.0	32.1
150	810	1499	8.03	71.6 *	10.5	101.7	29.5
180	765	1468	6.80	76.8	11.3	89.0	26.1
210	724	1236	6.67 *	62.2	11.6	100.9	26.1
240	661	1194	6.34 *	65.6	11.7	104.2	23.9
270	622 *	1124	5.41 *	58.6	11.6	93.9	25.4
300	580 *	959 *	5.44 *	66.6	12.0	122.7	22.6
**330**	552 *	848 *	5.03 *	52.6	11.1	118.1 *	22.9
360	574	964	4.47	68.8	11.8	125.4	24.7
375	521	935	4.98	**135.9 #**	11.2	109.0	22.8
390	523	913	4.68	**110.7 #**	11.7	126.3	23.0
405	**452 #**	846	3.88	**97.1 #**	12.4	112.0	22.0
420	**477 #**	748	3.89	**70.4 #**	**12.7 #**	106.5	23.1
450	**437 #**	790	**3.69 #**	82.8	**13.0 #**	96.5	23.8
480	**386 #**	**716 #**	**3.21 #**	71.0	**13.9 #**	115.3	26.1
510	**365 #**	730	**3.69 #**	80.5	**13.5 #**	122.3	**26.4 #**
540	**332 #**	**652 #**	**3.31 #**	**92.9 #**	**14.0 #**	137.7	26.0
570	**299 #**	**667 #**	**3.23 #**	75.3	**13.4 #**	127.8	24.9
			**Healthy Controls**				
**Time/min**	**Acetone / ppbV**	**2-Propanol /ppbV**	**Pentanal /ppbV**	**Ethanol / ppbV**	**dimethyl sulfide / ppbV**	**isoprene / ppbV**	**Limonene** **/ ppbV**
**baseline**	370	640	5.03	40.8	13.5	99.4	28.7
90	378	610	4.69	50.5	11.9 *	101.1	53.1 *
105	374	838	5.52	57.5	12.9 *	83.4	43.9 *
120	362	789	5.16	76.3	13.1 *	89.6	38.3 *
135	382	816	4.79	78.7 *	12.7	81.8	29.3
150	365	887	4.03	77.1 *	13.1	96.2 *	28.6
180	326	698	3.47 *	73.8	13.8	88.5 *	28.3
210	319 *	759	3.42 *	47.9	12.6	105.2	24.5
240	287 *	688	3.60 *	56.8	13.9	117.3	25.4
270	280 *	653	3.14 *	45.2	13.4	108.4	27.4
300	272 *	559 *	3.14 *	49.0	12.8	124.9	26.6
**330**	273 *	580	2.87 *	50.1	12.6	127.6	25.8
360	273	553	3.54	49.8	12.5	110.5	24.2
375	260	590	3.44	**95.6 #**	12.8	**105.3 #**	24.3
390	259	661	3.26	**105.6 #**	13.5	122.6	24.6
405	256	599	2.82	**111.4 #**	13.3	**113.7 #**	24.0
420	**248 #**	510	2.71	64.2	13.8	**118.0 #**	24.0
450	**232 #**	442	2.37	64.5	**14.0 #**	122.0	23.4
480	**224 #**	**432 #**	**2.46 #**	48.4	**14.4 #**	132.5	24.2
510	**227 #**	439	**2.30 #**	45.6	**14.3 #**	131.1	24.1
540	**217 #**	**421 #**	**2.24 #**	68.1	**15.0 #**	129.6	24.6
570	**210 #**	**458 #**	**2.18 #**	51.4	**14.7 #**	142.0	**24.4 #**

**Table 4:** Pearson product correlation coefficients between distinct VOCs and CGM as well as serum parameters (each *p* < 0.001). Detailed data are given in Supplementary Tables S3 and S4.

		Acetone	2-Propanol	Pentanal
sLepR				
	overall	0.561	0.487	0.542
	T1DM patients	0.364	0.321	0.424
	controls	0.500	0.321	0.374
Blood Glucose				
	overall	0.697	0.624	0.566
	T1DM patients	0.610	0.553	0.468
	controls	0.331	0.327	0.304
CGM data				
	overall	0.686	0.622	0.565
	T1DM patients	0.591	0.557	0.467
	controls	0.331	0.327	0.304

## Supplementary Materials

The following are available online at https://www.mdpi.com/2077-0383/8/11/1797/s1, Table S1: Cross-sectional comparison (healthy controls vs T1DM patients) of serum parameters at each time point via Mann-Whitney rank sum tests or t-tests in case of normal distribution; Table S2: Cross-sectional comparison (healthy controls vs T1DM patients) of VOC concentrations at each time point of blood drawing via Mann-Whitney rank sum tests or t-tests in case of normal distribution.; Table S3: Pearson product moment correlation analysis between VOCs and serum parameters.; Table S4: Pearson product moment correlation analysis between VOCs and interstitial glucose concentrations.
