# Supplementary material for: Non-Invasive Assessment of Metabolic Adaptation in Paediatric Patients Suffering from Type 1 Diabetes Mellitus
Source: J Clin Med. 2019 Oct 26;8(11):1797. doi: 10.3390/jcm8111797 (PMC6912469; doi:10.3390/jcm8111797)
Supplement: Supplementary file 1 [file jcm-08-01797-s001.pdf]

Table S1 Cross-sectional comparison (healthy controls vs T1DM patients) of serum parameters at each time point via Mann-Whitney rank sum tests or t-tests in case of normal distribution (marked with an asterisk). A p-value of 0.05 or lower was considered statistically significant. The table contains the p-values. Statistically significant changes are marked in bold.

**Time / min**	**glucose**	**insulin**	**glucagon**	**Tri-glycerides**	**cholesterol**	**leptin**	**sLepR**
**Baseline (60)**	**<0.001**	**0.013**	0.835	0.222	0.059	0.275	**<0.001**
**90**	**<0.001**	0.065	0.099	0.100	**0.038**	0.445	**<0.001**
**105**	**<0.001**	0.307	**0.021**	0.076	**0.049**	0.217	**<0.001**
**120**	**<0.001**	**<0.001**	0.085	0.121	0.091	0.230	**<0.001**
**135**	**<0.001**	**<0.001**	0.089	0.051	**0.021**	0.204	**<0.001**
**150**	**<0.001**	**<0.001**	0.154	0.071	0.096	0.289	**<0.001**
**180**	**<0.001**	**<0.001**	0.292	**0.050**	**0.028**	0.390	**<0.001**
**210**	**<0.001**	**<0.001**	0.118	**0.038**	0.076	0.351	**<0.001**
**240**	**<0.001**	**<0.001**	0.049	0.084	0.054	0.472	**<0.001**
**270**	**<0.001**	**<0.001**	0.146	0.113	**0.009**	0.43	**<0.001**
**300**	**<0.001**	**<0.001**	**0.030**	0.098	**0.045**	0.316	**<0.001**
**330**	**<0.001**	**<0.001**	**0.040**	0.130	**0.009**	0.352	**<0.001**
**360**	**<0.001**	0.379	0.183	**0.020**	**0.003**	0.390	**<0.001**
**375**	**<0.001**	**0.002**	0.098	**0.014**	**0.022**	0.532	**<0.001**
**390**	**<0.001**	**<0.001**	**0.020**	**0.004**	0.067	0.429	**<0.001**
**405**	**<0.001**	**<0.001**	0.087	**0.005**	**0.016**	0.472	**<0.001**
**420**	**<0.001**	**<0.001**	**0.034**	**0.037**	0.082*	0.564	**<0.001**
**450**	**0.004**	**<0.001**	0.090	**0.038**	**0.013***	0.541	**<0.001**
**480**	**0.040**	**<0.001**	**0.009***	0.091	**0.037***	0.661	**<0.001**
**510**	**0.018**	**<0.001**	**0.015***	**0.032**	**0.017***	0.445	**<0.001**
**540**	**<0.001**	**<0.001**	0.112	0.058	0.174	0.337	**<0.001**
**570**	**<0.001**	**<0.001**	**0.003***	**0.009**	0.159	0.192	**<0.001**

Table S2 Cross-sectional comparison (healthy controls vs T1DM patients) of VOC concentrations at each time point of blood drawing via Mann-Whitney rank sum tests or t-tests in case of normal distribution (marked with an asterisk). A p-value of 0.05 or lower was considered statistically significant. The table contains the p-values. Statistically significant changes are marked in bold.

**Time / min**	**acetone**	**2-propanol**	**pentanal**	**ethanol**	**Dimethyl sulfide**	**isoprene**	**limonene**
**Baseline (60)**	**<0.001**	**<0.001**	**0.001**	0.778	0.693*	0.953	0.334*
**90**	**<0.001**	**<0.001**	**0.009**	0.418	0.916	0.549	0.699
**105**	**<0.001**	**<0.001**	**<0.001**	0.549	0.824	0.227	0.460
**120**	**<0.001**	**<0.001**	**<0.001**	0.647	0.812*	0.805	0.953
**135**	**<0.001**	**<0.001**	**<0.001**	0.734	0.953	0.879	0.769
**150**	**<0.001**	**<0.001**	**<0.001**	0.630	0.991	0.581	0.842
**180**	**<0.001**	**<0.001**	**<0.001**	0.460	0.879*	0.489	0.342
**210**	**<0.001**	**<0.001**	**<0.001**	0.098	0.972	0.991	0.972
**240**	**<0.001**	**<0.001**	**<0.001**	0.227	0.836*	0.935	0.769
**270**	**<0.001**	**<0.001**	**0.001**	0.526	0.751	0.787	0.661*
**300**	**<0.001**	**<0.001**	**<0.001**	0.265	1.000	0.644*	0.581
**330**	**<0.001**	**<0.001**	**0.007**	0.851	0.860	0.370*	0.734
**360**	**<0.001**	**<0.001**	**0.042**	0.213	0.824	0.405	0.769
**375**	**<0.001**	**0.002**	**0.053**	0.275	0.935	0.432	0.699
**390**	**<0.001**	**0.003**	**0.018**	0.716	0.991	0.392	0.916
**405**	**<0.001**	**0.002**	**0.007**	0.699	0.860	0.265	0.751
**420**	**<0.001**	**<0.001**	**0.013**	0.699	0.787	0.664	0.879
**450**	**<0.001**	**0.006**	**0.011**	0.432	0.935	0.769	0.716
**480**	**<0.001**	**0.003**	**0.017**	0.432	0.734	0.751	0.489
**510**	**<0.001**	**0.003**	**0.002**	**0.045**	0.842	0.565	0.354
**540**	**<0.001**	**0.002**	**<0.001**	**0.021***	0.972	0.534	0.330
**570**	**<0.001**	**0.002***	**0.009**	**0.040**	0.824	0.534	0.330

Table S3 Pearson product moment correlation analysis between VOCs and serum parameters. Correlations that were significant on the 0.05 level (of p-values) are marked with an asterisk (*). Correlations that were significant on the 0.01 level (of p-values) are marked with two asterisks (**).

Both groups n = 968		blood glucose	cholesterol	triglyceride	leptin	sLepR	insulin	glucagon
**acetone**	Pearson Correlation	0.697**	0.269**	0.191**	0.028	0.561**	0.164**	0.022
	p	<0.001	<0.001	<0.001	0.389	<0.001	<0.001	0.498
**2-propanol**	Pearson Correlation	0.624**	0.209**	0.159**	0.099**	0.487**	0.152**	0.047
	p	<0.001	<0.001	<0.001	0.002	<0.001	<0.001	0.141
**pentanal**	Pearson Correlation	0.566**	0.247**	0.216**	0.001	0.542**	0.132**	0.008
	p	<0.001	<0.001	<0.001	0.980	<0.001	<0.001	0.807
**ethanol**	Pearson Correlation	0.119**	0.074*	0.035	0.198**	0.049	0.025	0.063
	p	<0.001	0.021	0.277	<0.001	0.125	0.432	0.051
**Dimethyl sulfide**	Pearson Correlation	0.016	0.244**	0.304**	0.195**	0.018	0.033	0.114**
	p	0.628	<0.001	<0.001	<0.001	0.571	0.311	<0.001
**isoprene**	Pearson Correlation	0.018	0.085**	0.037	0.294**	0.0.027	0.031	0.009
	p	0.573	0.008	0.249	<0.001	0.394	0.337	0.772
**limonene**	Pearson Correlation	0.091**	0.169**	0.107**	0.083**	0.087**	0.071*	0.065*
	p	0.005	<0.001	0.001	0.010	.007	0.028	0.043
**T1DM Group n = 484**		**blood glucose**	**cholesterol**	**triglyceride**	**leptin**	**sLepR**	**insulin**	**glucagon**
**acetone**	Pearson Correlation	0.610**	0.199**	0.162**	0.076	0.364**	0.026	0.191**
	p	<0.001	<0.001	<0.001	0.093	<0.001	0.570	<0.001
**2-propanol**	Pearson Correlation	0.553**	0.118**	0.102*	0.148**	0.321**	0.008	0.143**
	p	<0.001	0.010	0.025	0.001	<0.001	0.855	0.002
**pentanal**	Pearson Correlation	0.468**	0.190**	0.190**	0.038	0.424**	0.032	0.153**
	p	<0.001	<0.001	<0.001	0.403	<0.001	0.476	0.001
**ethanol**	Pearson Correlation	0.067	0.220**	0.109*	0.245**	0.052	0.021	0.055
	p	0.142	<0.001	0.016	<0.001	0.251	0.650	0.228
**Dimethyl sulfide**	Pearson Correlation	0.079	0.355**	0.315**	0.097*	0.124**	0.004	0.135**
	p	0.081	<0.001	<0.001	0.033	0.006	0.931	0.003
**isoprene**	Pearson Correlation	0.174**	0.175**	0.023	0.396**	0.083	0.091*	0.113*
	p	<0.001	<0.001	0.621	<0.001	0.067	0.045	0.013
**limonene**	Pearson Correlation	0.175**	0.160**	0.077	0.208**	0.076	0.100*	0.082
	p	<0.001	<0.001	0.090	<0.001	0.095	0.028	0.073

Control Group n = 484		blood glucose	cholesterol	triglyceride	leptin	sLepR	insulin	glucagon
**acetone**	Pearson Correlation	0.281**	0.065	0.291**	0.451**	0.500**	0.023	0.067
	p	<0.001	0.156	<0.001	<0.001	<0.001	0.611	0.140
**2-propanol**	Pearson Correlation	0.246**	0.074	0.134**	0.423**	0.321**	0.029	0.145**
	p	<0.001	0.103	0.003	<0.001	<0.001	0.532	0.001
**pentanal**	Pearson Correlation	0.321**	0.085	0.135**	0.301**	0.374**	0.132**	0.031
	p	<0.001	0.062	0.003	<0.001	<0.001	0.004	0.502
**ethanol**	Pearson Correlation	0.244**	0.023	190*0*	0.197**	0.100*	0.011	0.227**
	p	<0.001	0.607	<0.001	<0.001	0.029	0.818	<0.001
**Dimethyl sulfide**	Pearson Correlation	0.001	0.168**	0.365**	0.491**	0.360**	0.261**	0.054
	p	0.987	<0.001	<0.001	<0.001	<0.001	<0.001	0.237
**isoprene**	Pearson Correlation	0.102*	0.156**	0.059	0.079	0.094*	0.114*	0.103*
	p	0.026	0.001	0.196	0.084	0.038	0.012	0.023
**limonene**	Pearson Correlation	0.121**	0.202**	0.158**	0.139**	0.329**	0.066	0.065
	p	0.008	<0.001	0.001	0.002	<0.001	0.148	0.157

Table S4 Pearson product moment correlation analysis between VOCs and interstitial glucose concentrations. Correlations that were significant on the 0.05 level (of p-values) are marked with an asterisk (*). Correlations that were significant on the 0.01 level (of p-values) are marked with two asterisks (**).

		**Both groups n = 4107**	**T1DM Group n = 2010**	**Control Group n = 2097**
**ethanol**	Pearson Correlation	0.112**	0.061**	0.296**
	p	<0.001	0.007	<0.001
**acetone**	Pearson Correlation	**0.686****	**0.591****	0.331**
	p	<0.001	<0.001	<0.001
**2-propanol**	Pearson Correlation	**0.622****	**0.557****	0.327**
	p	<0.001	<0.001	<0.001
**Dimethyl sulfide**	Pearson Correlation	0.064**	0.140**	0.056*
	p	<0.001	<0.001	0.010
**isoprene**	Pearson Correlation	0.036*	0.191**	0.094**
	p	0.020	<0.001	<0.001
**pentanal**	Pearson Correlation	**0.565****	**0.467****	0.304**
	p	<0.001	<0.001	<0.001
**limonene**	Pearson Correlation	0.052**	0.083**	0.093**
	p	0.001	<0.001	<0.001
